# Corrigendum: Emodin Interferes With AKT1-Mediated DNA Damage and Decreases Resistance of Breast Cancer Cells to Doxorubicin

**DOI:** 10.3389/fonc.2021.687841

**Published:** 2021-06-02

**Authors:** Bo Li, Xin Zhao, Lei Zhang, Wen Cheng

**Affiliations:** ^1^ Department of Ultrasound, Harbin Medical University Cancer Hospital, Harbin, China; ^2^ Department of Orthopedics, The Second Affiliated Hospital of Harbin Medical University, Harbin, China; ^3^ Department of Ultrasound, The Second Affiliated Hospital of Harbin Medical University, Harbin, China

**Keywords:** AKT1, DNA repair, bioinformatics, breast cancer, emodin

In the original article, there was a mistake in **Figure 7C** as published. The images in **Figure 7C** are incorrectly assembled due to incorrect naming of the image files. The corrected **Figure 7** appears below.

**Figure 7:**
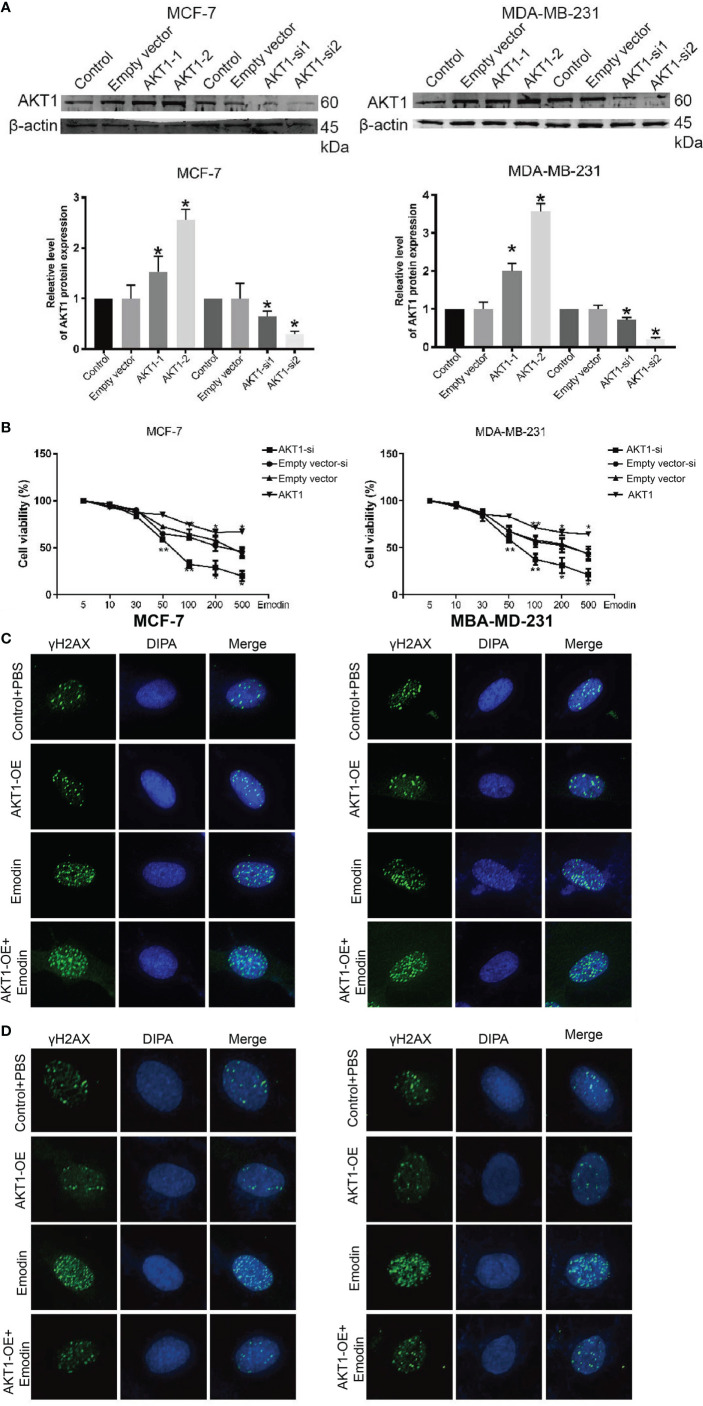
BC cells complemented with AKT-1-si or AKT-1-oe regulated to Emodin. **(A)** The expression of AKT1 after treatment with AKT1-siRNA or AKT1 overexpression. **(B)** CCK-8 assay shows the BC cells complemented with AKT1-siRNA or AKT1 over-expression. **(C)** DNA damage assays shows that the BC cells complemented with AKT1-siRNA promote Emodin compared with cells treated with AKT1-siRNA or Emodin alone. **(D)** DNA damage assays shows that the BC cells complemented with AKT1 over-expression resists Emodin compared with cells treated with AKT1-overexpression or Emodin alone. The analysis was conducted using the Student’s t-test. p > 0.05, **p < 0.01, *p < 0.05.

The authors apologize for this error and state that this does not change the scientific conclusions of the article in any way. The original article has been updated.

